# Ubiquitin-specific protease 4 promotes hepatocellular carcinoma progression via cyclophilin A stabilization and deubiquitination

**DOI:** 10.1038/s41419-017-0182-5

**Published:** 2018-02-02

**Authors:** Tianyi Li, Bin Yan, Yang Ma, Junyong Weng, Shouwen Yang, Nan Zhao, Xiaoliang Wang, Xing Sun

**Affiliations:** 1 Department of General Surgery, Shanghai General Hospital, Shanghai Jiao Tong University, School of Medicine, Shanghai, China; 2 Department of General Surgery, Ruijin Hospital, Shanghai Jiao Tong University, School of Medicine, Shanghai, China; 3 Department of Gynaecology, International Peace Maternity and Child Health Hospital, Shanghai Jiao Tong University, School of Medicine, Shanghai, China; 4 Department of General Surgery, Shanghai General Hospital, Nanjing Medical University, Shanghai, China

## Abstract

Ubiquitin-specific protease 4 (USP4) is a member of the deubiquitinating enzyme family, which plays an important role in human tumor diseases. However, the mechanisms by which USP4 facilitates tumor development, especially in hepatocellular carcinoma (HCC), remain unclear. Clinically, we found that USP4 is overexpressed in human HCC tissues compared with adjacent non-tumoral tissues and is significantly correlated with malignant phenotype characteristics, including tumor size, tumor number, differentiation, serum alpha-fetoprotein level, and vascular invasion. Moreover, Kaplan–Meier survival analysis showed a poor overall survival rate in patients with USP4-overexpressing tumors. Analyses of univariate and multivariate Cox proportional hazard models indicated that USP4 is a prognostic biomarker for poor outcome. Using in vitro and in vivo assays, we demonstrated that USP4 overexpression enhanced HCC cell growth, migration, and invasion. Mechanistically, cyclophilin A (CypA) was identified as an important molecule for USP4-mediated oncogenic activity in HCC. We observed that USP4 interacted with CypA and inhibited CypA degradation via deubiquitination in HCC cells. Subsequently, the USP4/CypA complex activated the MAPK signaling pathway and prevented CrkII phosphorylation. These data suggest that USP4 acts as a novel prognostic marker, offering potential therapeutic opportunities for HCC.

## Introduction

Liver cancer is the sixth most frequently diagnosed cancer, with nearly 800,000 deaths each year worldwide, and is more common in less developed countries^1^. Hepatocellular carcinoma (HCC), which accounts for approximately 90% of all cases of primary liver cancer, is one of the leading causes of cancer-related deaths worldwide, with a continuously rising incidence^2^. In 2015, the incidence and mortality rates of HCC in China ranked fourth and third among tumor diseases, respectively^3^. Although advanced treatments are currently available, the overall survival (OS) rate of HCC patients has not improved, mainly due to the high rate of recurrence and metastasis. Identification of specific genetic alterations and biomarkers related to HCC may facilitate earlier diagnosis and treatment.

Alterations in cancer-related gene expression are considered to contribute to carcinogenesis because of their effects on cell biological functions, such as proliferation, cell–cell adhesion, and motility. Some oncogenes and tumor suppressor genes have been described in HCC development. For example, PEG10 was found to be associated with poor survival and recurrence in HCC patients, and ARID2 acts as a tumor suppressor that inhibits tumor metastasis in HCC cells^4, 5^. However, the protein products and their post-translational modifications, including ubiquitination, always determine the biological functions of these genes. Thus, identification of novel regulation mechanisms of these genes at the protein level may potentially be a subject of significant interest for HCC treatment.

Ubiquitin, a 76-amino acid protein, is attached to target proteins and regulates protein half-life, localization, and activity. Protein ubiquitination and the reverse process, deubiquitination, are significant post-translational modifications that regulate diverse cellular processes, such as cell growth, proliferation, DNA damage repair, and apoptosis^6^. Deubiquitination is mediated by deubiquitinating enzymes (DUBs), and the nearly 100 known DUBs can be divided into five families^7^. Among them, ubiquitin-specific proteases (USPs) constitute the largest subclass of DUBs, with more than 60 members^8^. Some USPs have been found to be closely related to cancer progression^9, 10^. However, many questions remain concerning the mechanism of USPs in cancers.

Ubiquitin-specific protease 4 (USP4), a member of the USPs family, has been associated with many human malignant tumors, including colorectal cancer^11^, breast cancer,^12^ and liver cancer^13^. Diverse biological functions of USP4 have been reported in different studies. USP4 may have oncogenic properties through positive regulation of the WNT/β-catenin pathway via deubiquitination and stabilization of β-catenin in colorectal cancer^14^. HDAC2 and TAK1 have also been reported to be deubiquitinated by USP4, resulting in p53 suppression and inhibition of nuclear factor-κB (NF-κB) activity^15, 16^. However, the relevant roles of USP4 in HCC have not been well established and require further exploration.

In this study, we examined USP4 expression levels in HCC clinical tissue samples and cell lines. The effects of USP4 on biological functions in HCC cells were assessed in vitro and in vivo. Finally, co-immunoprecipitation (Co-IP) and quantitative proteomics analyses were used to investigate a USP4 partner protein to explore the mechanism of USP4 in HCC development.

## Results

### USP4 is overexpressed in HCC tissues and predicts poor clinical outcomes in HCC patients

**Fig. 1:**
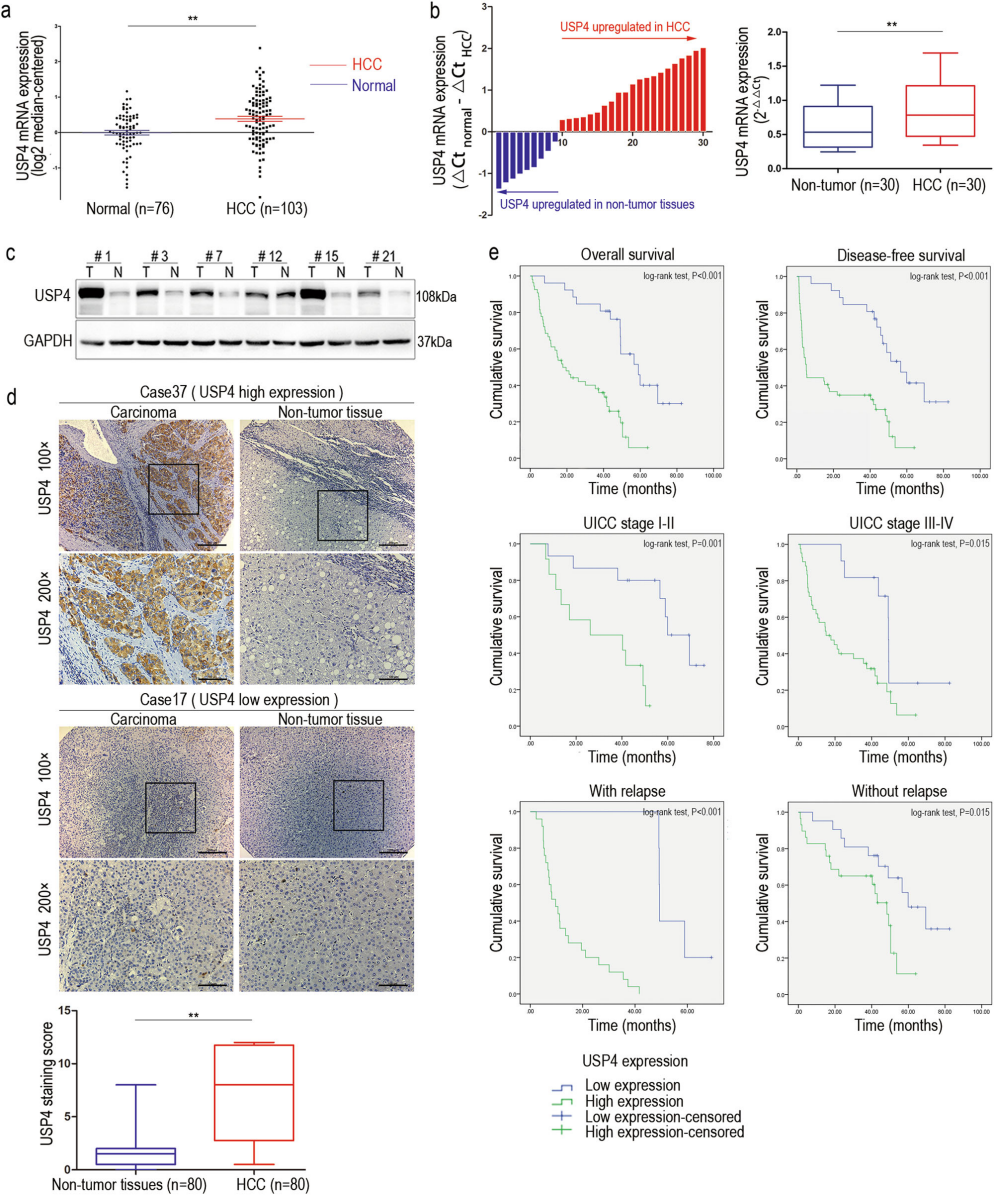
High expression of USP4 in HCC tissues and clinical significance. **a** USP4 mRNA levels in microarray datasets from Oncomine Database, Chen Liver. **b** qRT-PCR detection of USP4 expression in 30 paired specimens of HCC tissues and matched adjacent non-tumorous liver tissues. **c** Western blotting analysis of USP4 expression in six paired specimens randomly selected. T HCC tissues; N corresponding normal tissues. GAPDH was used as the loading control. **d** IHC analysis and staining scores of USP4 expression in 80 pairs of HCC and matched non-tumor tissues. Representative images of different USP4 expression levels are shown. Scale bars ×100: 200μm, Scale bars ×200: 100μm. **e** Kaplan–Meier analysis with log-rank testing of survival was performed in HCC patients with different USP4 expression levels. Error bars represent the SD. *P < 0.05 and **P < 0.01

We first analyzed a public gene expression array database from the Oncomine Database to explore the USP4 expression level in human HCC and normal liver tissues^17^. A much higher expression level of USP4 messenger RNA (mRNA) was found in HCC than in normal liver tissues (*P* < 0.01) (Fig. 1a). Subsequently, thirty paired specimens were selected to explore the USP4 expression level in HCC and matched non-tumor tissues using quantitative real-time PCR (qRT-PCR). Twenty-one cases (70%) of HCC tissues had higher USP4 transcript levels than matched surrounding tissues (*P* < 0.01) (Fig. 1b). Western blotting confirmed the results and showed that USP4 protein levels were upregulated in HCC tissues compared with the level in the corresponding normal tissues (Fig. 1c). Next, immunohistochemical (IHC) staining of USP4 in a tissue microarray that included 80 pairs of HCC and matched non-tumor tissues was performed. We observed USP4 expression in the cytoplasm and divided the 80 HCC patients into USP4 high-expression (54/80) and low-expression (26/80) groups according to the staining intensity. The results confirmed that USP4 is overexpressed in tumor tissues (*P* < 0.01) (Fig. 1d). The relationship between USP4 expression and clinicopathological characteristics was then investigated **(**Table 1**)**. The results showed that increased USP4 expression was highly correlated with histological differentiation (*P* = 0.002), tumor size (*P* = 0.003), tumor number (*P* = 0.043), vascular invasion (*P* = 0.003), and serum alpha-fetoprotein (AFP) levels (*P* = 0.024). However, no significant correlations were found between USP4 expression and age, gender, tumor encapsulation, liver cirrhosis, hepatitis B surface antigen, serum alanine aminotransferase, or total bilirubin levels (*P* > 0.05 for all).

**Table 1:** USP4 expression and clinicopathological characteristics in HCC (*n* = 80)

	USP4 expression		
	Low (26)	High(54)	P*
Sex			
Male	23	45	0.789
Female	3	9	
Age, years			
≤50	12	28	0.633
>50	14	26	
Tumor differentiation			
I–II	15	12	0.002
III–IV	11	42	
Tumor encapsulation			
Present	20	32	0.121
Absent	6	22	
Tumor size, cm			
≤5	19	20	0.003
>5	7	34	
Tumor number			
Single	17	46	0.043
Multiple	9	8	
Vascular invasion			
No	19	20	0.003
Yes	7	34	
Liver cirrhosis			
No	8	29	0.061
Yes	18	25	
HBsAg			
−	2	6	0.937
+	24	48	
Serum AFP, ng/mL			
≤20	11	10	0.024
>20	15	44	
Serum ALT, U/L			
≤75	23	50	0.849
>75	3	4	
Serum TB, μmol/L			
≤17	16	42	0.128
>17	10	12	

^*^Pearson’s chi-square test was used. *P* < 0.05 was considered statistically significant

Subsequently, Kaplan–Meier survival analysis with log-rank testing was used to determine the relationship between USP4 and OS and disease-free survival (DFS). Patients with high USP4-expressing tumors had a significantly lower OS and DFS rate than those with low USP4-expressing tumors (*P* < 0.01 for both). The median OS and DFS time of HCC patients with low USP4 expression was 58.9 and 56.6 months, respectively, compared with 17.8 and 5.1 months in the high USP4 expression group, suggesting that high USP4 expression is a prognostic indicator for HCC patients. We observed a similar trend in which patients with high USP4 expression had a shorter OS time than those with low expression when further OS analysis was performed based on the UICC stage and tumor relapse (Fig. 1e). The Cox proportional hazards model was used for univariate and multivariate analysis of OS and postoperative cumulative recurrence (Table 2). In univariate analysis of OS and cumulative recurrence, USP4 expression, serum AFP level, vascular invasion, and tumor size emerged as significant independent prognostic factors (*P* < 0.05 for all). Interestingly, we found that tumor differentiation acts as a prognostic factor in OS univariate analysis (*P* = 0.011) but not in the cumulative recurrence univariate analysis (*P* = 0.078). Then, multivariate analysis revealed that USP4 expression, vascular invasion, and tumor size (*P* < 0.05 for all) were independent prognostic factors for OS and cumulative recurrence. Collectively, these results indicate that high USP4 expression is likely involved in HCC progression and may act as an independent prognostic biomarker for poor outcomes in patients with HCC.

**Table 2:** Univariate and multivariate Cox proportional hazards model for overall survival and postoperative cumulative recurrence

	Overall survival				Cumulative recurrence			
	Univariate		Multivariate		Univariate		Multivariate	
Factors	HR (95% CI)	*P-*value	HR (95% CI)	*P*-value	HR (95% CI)	*P-*value	HR (95% CI)	*P*-value
Sex (female vs. male)	0.507 (0.202−1.271)	0.147	−	−	0.575 (0.174−1.895)	0.363	−	−
Age, years (>50 vs. ≤50)	0.627 (0.378−1.066)	0.085	−	−	0.616 (0.297−1.278)	0.193	−	−
Tumor differentiation (III/IV vs. I/II)	2.140 (1.194−3.835)	0.011	1.300 (0.682−2.479)	0.425	2.094 (0.919−4.768)	0.078		
Tumor encapsulation (absent vs. present)	0.957 (0.551−1.662)	0.877	−	−	1.295 (0.622−2.695)	0.489	−	−
Tumor size (diameter, cm) (>5 vs. ≤5)	5.483 (3.002−10.014)	<0.001	3.412 (1.733−6.718)	<0.001	7.086 (2.778−18.069)	<0.001	4.119 (1.491−11.380)	0.006
Tumor number (multiple vs. single)	1.597 (0.893−2.856)	0.114	−	−	1.126 (0.481−2.639)	0.784	−	−
Vascular invasion (yes vs. no)	4.915 (2.769−8.724)	<0.001	4.554 (2.429−8.536)	<0.001	10.266 (3.829−27.522)	<0.001	8.706 (2.977−25.458)	<0.001
Liver cirrhosis (yes vs. no)	0.763 (0.452–1.289)	0.312	−	−	0.697 (0.339−1.433)	0.327	−	−
HBsAg (+ vs. −)	1.083 (0.463−2.534)	0.853	−	−	0.805 (0.280−2.317)	0.687	−	−
Serum AFP, ng/mL (>20 vs. ≤20)	2.739 (1.425−5.262)	0.002	1.403 (0.649−3.033)	0.389	3.900 (1.340−11.355)	0.013	2.168 (0.656−7.157)	0.204
Serum ALT, U/L (>75 vs. ≤75)	2.062 (0.926−4.594)	0.077	−	−	1.860 (0.646−5.361)	0.250	−	−
Serum TB, μmol/L (>17 vs. ≤17)	0.585 (0.311−1.099)	0.095	−	−	0.490 (0.198−1.212)	0.122	−	−
USP4 expression (high vs. low)	4.076 (2.106−7.887)	<0.001	3.099 (1.510−6.357)	0.002	4.802 (1.760−13.099)	0.002	3.354 (1.123−10.031)	0.030

*CI* confidence interval, *HR* hazard ratio

### USP4 accelerates HCC cell proliferation, migration, and invasion in vitro

**Fig. 2:**
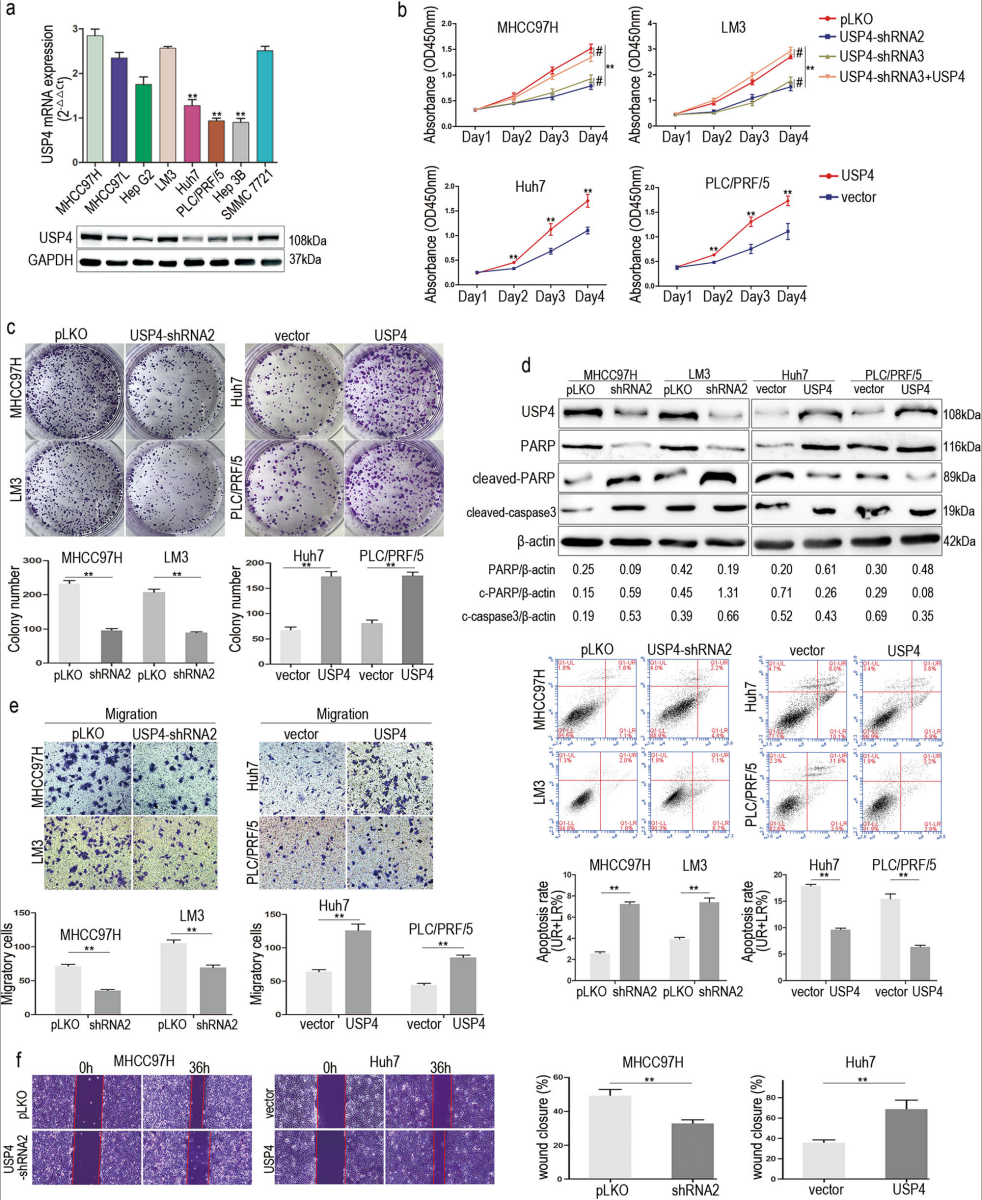
High USP4 expression promoted HCC cells growth and migration in vitro. **a** qRT-PCR and western blotting assays were used to detect the USP4 expression in eight HCC cell lines. The statistical analyses compared Huh7, PLC/PRF/5, and Hep3B cell lines with other HCC cell lines by using one-way ANOVA. **b** Cell proliferation in HCC cells with regulated USP4 expression was assessed by using CCK-8 assays. **c** Colony formation assays were performed in HCC cells with different levels of forced USP4 expression. The number of colonies was quantified. **d** Apoptosis in HCC cells with forced USP4 expression was assessed using FCM. UR+LR percentage represents the apoptosis rate. Western blotting assays were used to investigate expression of cell apoptosis indicator proteins. c-PARP, cleaved-PARP. c-caspase3, cleaved-caspase3. **e** The in vitro migration abilities of HCC cells with different forced USP4 expression were assessed using transwell assays. Representative images are shown. **f** Wound healing assays were performed to evaluate the migration of HCC cells with different forced USP4 expression. Cells were cultured in FBS-free medium in the experimental period and wound closure percentage was calculated. Each experiment was repeated three times. Error bars represent the SD. The statistical analyses compared the experimental group and the control group. *P < 0.05, **P < 0.01 and #*P* > 0.05

We speculated that USP4 overexpression may act as a cancer promoter in HCC. To test this hypothesis, USP4 levels were first compared in eight different human HCC cell lines. The results showed that USP4 was differentially expressed in the various HCC cell lines at both the mRNA and protein level (Fig. 2a). Among them, the MHCC97H, SMMC7721, and LM3 cell lines expressed high levels of USP4, but Huh7, Hep3B, and PLC/PRF/5 cells had low USP4 expression levels. We next used three different shRNA constructs to knock down USP4 expression in MHCC97H and LM3 cells. Two shRNAs were selected for use in further assays on HCC cells. Cells transfected with pLKO vector served as negative controls (Supplementary Figure 1a). Cell Counting Kit-8 (CCK-8) assays were used to evaluate the proliferation ability of cells. The results showed that proliferation was significantly inhibited by USP4 knockdown in MHCC97H and LM3 cells (*P* < 0.01), and was rescued by co-transfection with a shRNA-resistant USP4 cDNA (*P* < 0.01) (Fig. 2b). Then, Huh7 and PLC/PRF/5 cells with low USP4 expression were transfected with USP4 plasmid (Supplementary Figure 1b). We observed that the proliferation potential was significantly enhanced in both transfected cell lines (*P* < 0.01) (Fig. 2b). Moreover, colony formation assays confirmed this phenomenon. USP4 reduction in MHCC97H and LM3 or overexpression in Huh7 and PLC/PRF/5 cells impaired or promoted, respectively, the clonogenic ability of cells compared with that of control cells (*P* < 0.01) (Fig. 2c and Supplementary Figure 2a). Similarly, USP4 knockdown resulted in an increase in the apoptosis rate of MHCC97H and LM3 cells and upregulated the protein expression of cleaved-caspase3 and cleaved-PARP, which are indicators of cell apoptosis. In contrast, the apoptosis rate and related proteins were reduced when USP4 was overexpressed in Huh7 and PLC/PRF/5 cells (*P* < 0.01) (Fig. 2d and Supplementary Figure 2b). We next assessed HCC cell migration and invasion ability. Transwell assays showed that USP4 knockdown inhibited cell migration and invasion in MHCC97H and LM3 cells. Moreover, USP4 upregulation promoted Huh7 and PLC/PRF/5 cell migration and invasion ability (*P* < 0.01 for all) (Fig. 2e and Supplementary Figure 2c). Wound healing assays produced similar results (Fig. 2f and Supplementary Figure 2d). The effects of the shRNAs on the biological function of MHCC97H and LM3 cells could be reversed by co-transfection with an shRNA-resistant USP4 cDNA. In summary, these data indicate that USP4 enhances HCC cell proliferation, migration, and invasion and suppresses HCC cell apoptosis in vitro.

### USP4 overexpression promotes HCC cell tumorigenesis in vivo

**Fig. 3:**
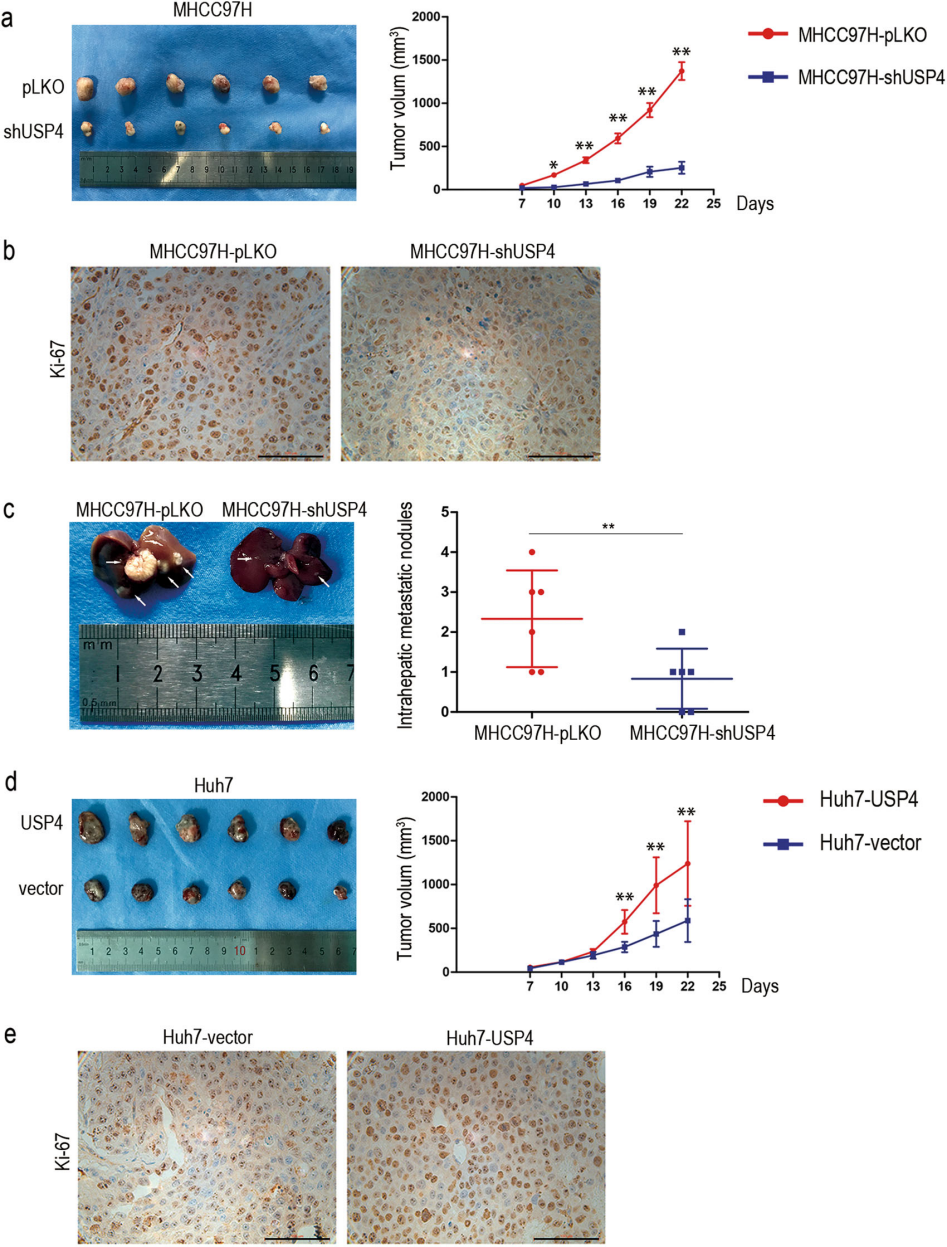
USP4 promoted HCC cell growth and intrahepatic metastasis in vivo. **a** USP4 knockdown suppressed HCC cell tumorigenesis in vivo (*n* = 6). The volume of subcutaneous tumors was measured. **b** IHC staining of xenograft tumors for the cell proliferation biomarker, Ki-67, derived from MHCC97H-shUSP4 cells and control cells. Representative images are shown. **c** Representative images of intrahepatic metastatic nodules derived from stable MHCC97H-shUSP4 cells and control cells are shown (*n* = 6). **d** USP4 overexpression promoted cell tumorigenesis in vivo (*n* = 6). **e** Representative images of IHC staining of xenograft tumors for Ki-67 derived from Huh7-USP4 cells and control cells are shown. Error bars represent the SD. *P < 0.05; **P < 0.01; ***P = 0.000. Scale bars: 100μm

Stable MHCC97H-shUSP4 cells and negative control group cells were successfully generated via lentivirus-mediated transduction. Nude mice were injected in the groin with the same number of MHCC97H-shUSP4 or control cells. Tumor sizes were measured every 3 days from Day 7 after implantation. The tumor volume measurements showed that MHCC97H-shUSP4 cells generated smaller subcutaneous xenografts in nude mice compared with the control cells (*P* < 0.05) (Fig. 3a). Using IHC staining, we found that tumor xenografts with USP4 knocked down showed lower Ki-67 expression, a cell proliferation marker, than controls (Fig. 3b). Next, MHCC97H-shUSP4 and control cells were inoculated into the hepatic lobes of nude mice. After 3 weeks, histological examination of the livers indicated that mice injected with USP4-down-regulated cells had fewer and smaller intrahepatic metastatic nodules than control group mice (*P* < 0.05) (Fig. 3c). However, neither group was found to develop lung metastases. To confirm this result, we performed a supplementary investigation in Huh7-USP4 stable overexpression cells and Huh7-control cells. Similar results were observed (Figs. 3d, e). Therefore, all these data demonstrate that high USP4 expression levels tend to facilitate HCC growth and intrahepatic metastasis in vivo.

### Cyclophilin A (CypA) is a potential USP4 complexation candidate according to proteomic analysis

**Fig. 4:**
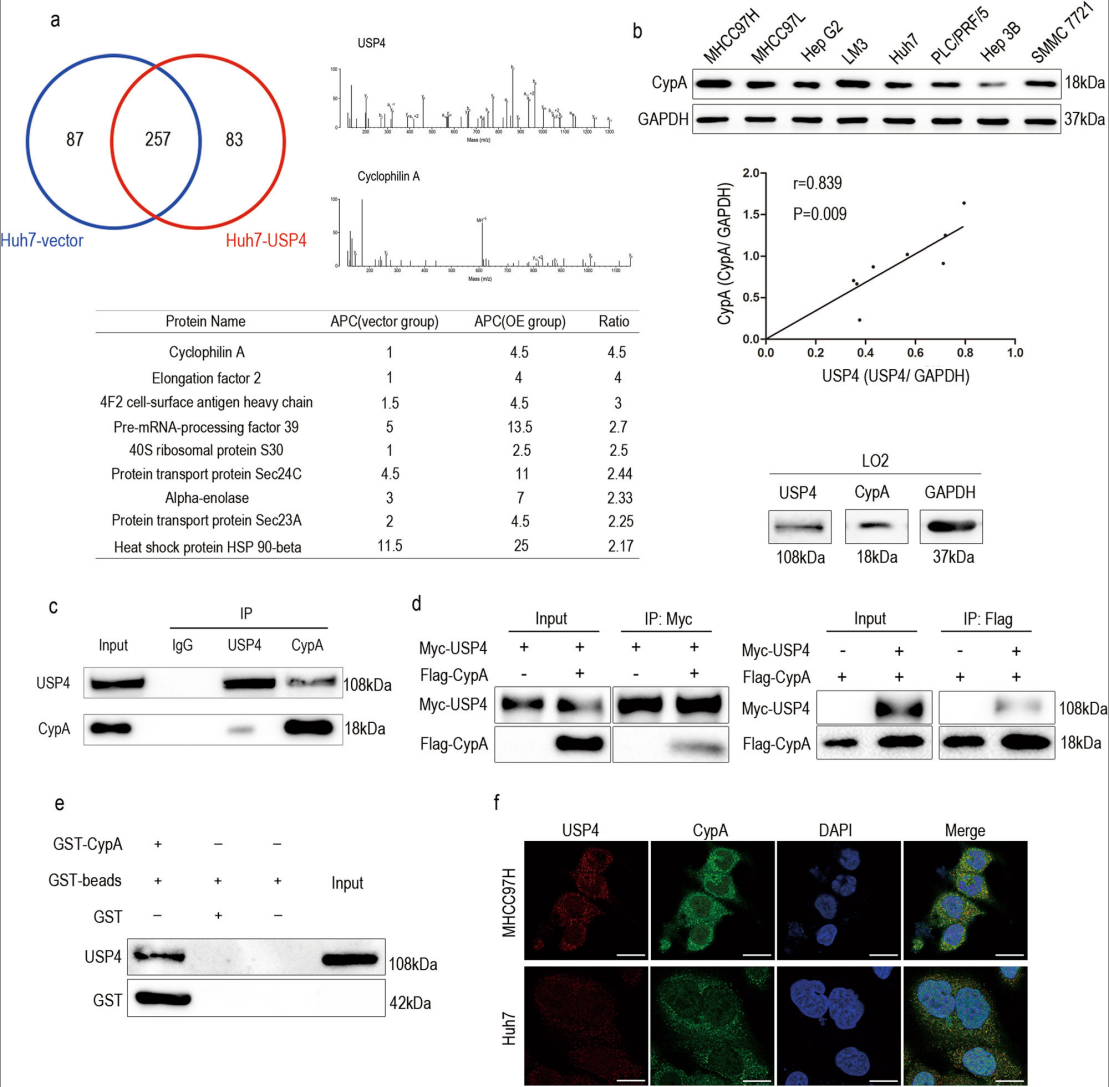
USP4 interacts with CypA. **a** Identification of USP4 binding partners using a combination of Co-IP and high-resolution LC-MS/MS analysis. The Venn diagram shows the number of binding partners of USP4 in the two groups. Nine overlapping proteins with a ratio of >2 are list. APC average peptide count, OE overexpression. **b** Western blotting assays were used to detect CypA expression in eight HCC cell lines and a normal liver cell line, LO2. Spearman correlation analysis was performed on the protein levels of USP4 and CypA in the eight HCC cell lines. **c** Western blotting analysis of lysates after Co-IP assays from MHCC97H cells to validate the endogenous formation of the USP4/CypA complex. **d** Western blotting analysis of lysates after Co-IP assays from 293T cells transfected with Myc-USP4 and Flag-CypA plasmids to validate exogenous USP4/CypA complex formation. **e** GST-CypA fusion protein was incubated with GST-beads and lysates of USP4 transfected 293T cells. The interacting USP4 was probed using western blotting. **f** Immunofluorescence staining was used to observe the expression of USP4 and CypA in MHCC97H and Huh7 cells. Representative images are shown. Scale bars: 10μm

Using Co-IP with antibody against USP4 and affinity enrichment followed by high-resolution LC-MS/MS analysis, quantitative proteomics analysis was performed to determine how USP4 influences biological functions in HCC cell lines. The Huh7 cell line, with low endogenous USP4 expression, was selected, and cells were divided into two groups, Huh7-USP4 and Huh7-vector. In total, 344 and 340 proteins were identified as the USP4 interactome in Huh7-vector and Huh7-USP4 cells, respectively. Among them, 257 proteins in the two cell lines examined were found to overlap. When the quantification ratio of >2 was set as the interactive protein threshold, nine proteins were identified as possibly showing increased interaction with USP4 when USP4 was overexpressed (Fig. 4a). Among them, CypA, with the highest quantification ratio of 4.5, was on the top of the list. In addition, CypA has been found to be upregulated in many human malignancies and may act as an oncogene^18, 19^. Therefore, we proposed that CypA plays an important role in USP4-mediated HCC progression. We further examined relationships between USP4 and CypA expression. First, the CypA protein levels in the same eight HCC cell lines were investigated using western blotting, and a positive correlation between USP4 and CypA was shown (*r* = 0.839 *P* = 0.009). LO2, a normal human liver cell line, was also selected to test the expression levels of USP4 and CypA using western blotting. Interestingly, we found both of the two proteins were lowly expressed in LO2 compared with HCC cell lines (Fig. 4b). Next, we investigated the biochemical relationship between USP4 and CypA proteins in cells. An endogenous Co-IP assay was performed in MHCC97H cells, and the results showed that endogenous USP4 forms a complex with endogenous CypA in MHCC97H cells (Fig. 4c). Then, Myc-USP4 and Flag-CypA were co-transfected into 293T cells, and reciprocal Co-IP assays were performed using antibodies against the Myc and Flag tags. The results revealed that Flag-CypA was co-immunoprecipitated with Myc-USP4 by the anti-Myc antibody. We obtained similar results showing that Myc-USP4 was precipitated using anti-Flag antibody (Fig. 4d). The Glutathione *S*-transferase (GST) pull-down assay also showed that USP4 could bind to purified GST-CypA in vitro (Fig. 4e). In addition, immunofluorescence staining demonstrated that USP4 and CypA co-localized primarily in the cytoplasm of MHCC97H and Huh7 cells (Fig. 4f).

Collectively, these data indicated that both endogenous and exogenous USP4 and CypA proteins can interact.

### USP4 stabilizes CypA protein and protects it from degradation through deubiquitination

As one of the DUBs, USP4 is involved in the post-translational modification of many proteins, which prompted us to investigate whether USP4 could regulate CypA expression.

**Fig. 5:**
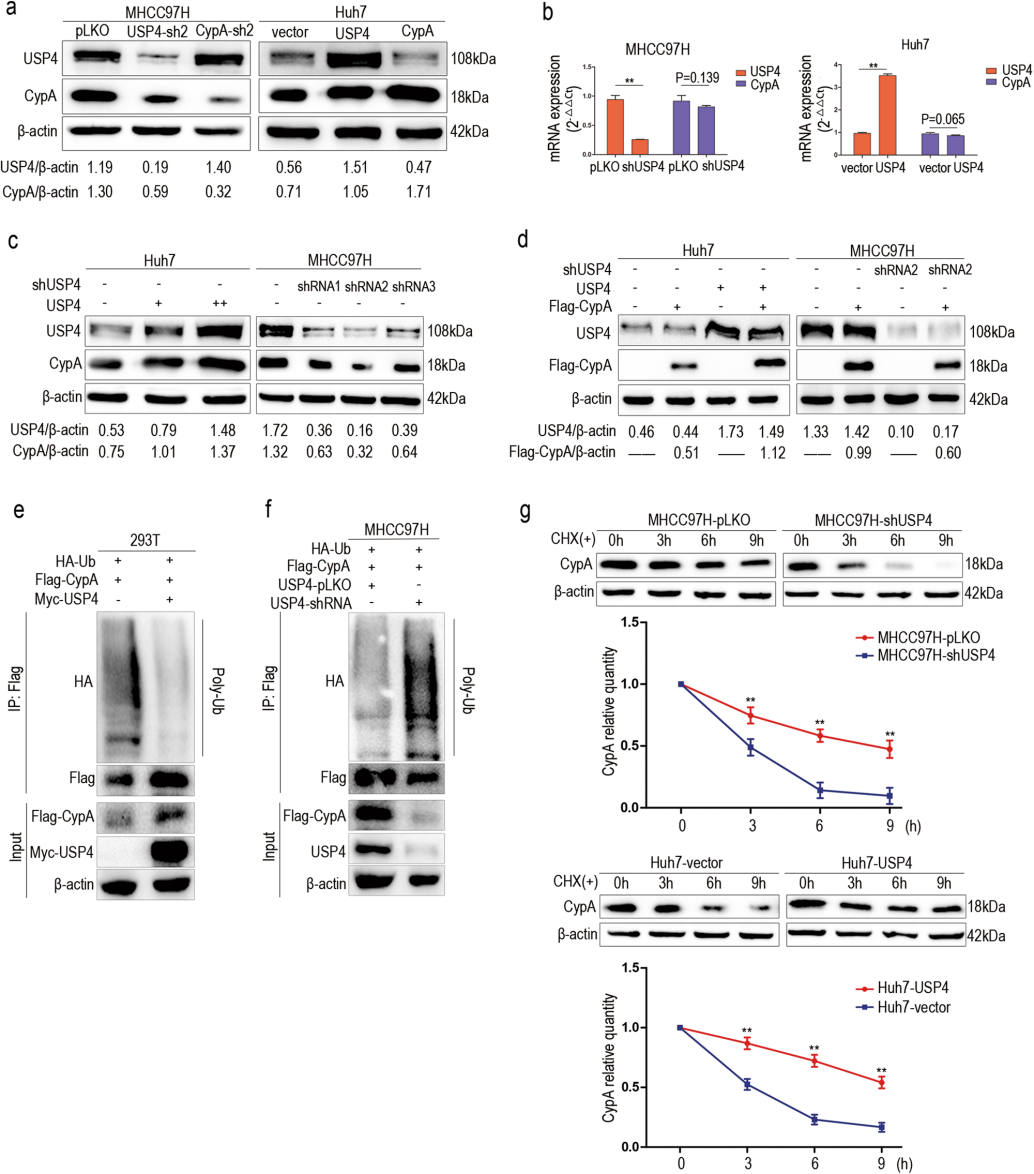
USP4 stabilizes and protects CypA from degradation via deubiquitination. **a** Different forced USP4 expression affected the protein levels of CypA, whereas regulated expression of CypA had no effect on USP4 protein levels. **b** CypA was not regulated by USP4 at the mRNA level. **c**, **d** USP4 overexpression increased CypA protein levels endogenously and exogenously in Huh7 cells. USP4 knockdown decreased CypA protein levels endogenously and exogenously in MHCC97H cells. **e** Impact of USP4 on CypA ubiquitination in vivo. Lysates of 293T cells were immunoprecipitated with an antibody against Flag-tag and analyzed using western blotting analyses to detect poly-ubiquitination of CypA. **f** The impact of USP4 on CypA ubiquitination in MHCC97H cells. Lysates of MHCC97H cells with USP4-shRNA transfected or not were immunoprecipitated with an antibody against Flag-tag and analyzed using western blotting analyses to detect poly-ubiquitination of CypA. **g** USP4 enhanced stability of the CypA protein. MHCC97H-pLKO/shUSP4 cells and Huh7-USP4/vector cells were exposed to CHX and collected at the indicated times. Expression of CypA was examined using western blotting assays. The experiments were repeated three times. Western blotting analyses were quantified via densitometry. Error bars represent the SD. **P < 0.01

Inhibition of USP4 by shRNA in MHCC97H cells significantly decreased CypA protein levels. Meanwhile, transfection of Huh7 cells with USP4 plasmid resulted in an increase in CypA expression. However, down- or upregulation of CypA in MHCC97H and Huh7 cells did not affect USP4 protein levels (Fig. 5a and Supplementary Figure 3a). Moreover, no significant change in CypA mRNA levels was detected using qRT-PCR after knockdown or upregulation of USP4 (Fig. 5b), demonstrating that USP4 regulates CypA expression at the protein level.

To further confirm this, different amounts of USP4 plasmid were transfected into Huh7 cells. Western blotting showed that endogenous CypA protein was significantly increased by USP4 overexpression in a dose-dependent manner. In contrast, down-regulation of USP4 via shRNAs in MHCC97H cells significantly decreased endogenous CypA (Fig. 5c). When testing the effect of USP4 on exogenous CypA levels, the results showed that Flag-CypA was upregulated after transfection with USP4 plasmid in Huh7 cells, and was significantly decreased due to down-regulation of USP4 via shRNAs in MHCC97H cells (Fig. 5d **and** Supplementary Figure 3b).

Because ubiquitination modification is an important path for protein degradation, we supposed that USP4 regulates the stability of its binding partner CypA through deubiquitination. To test this presumption, we performed an in vivo ubiquitination assay. Flag-CypA and HA-ubiquitin plasmids were co-transfected into 293T cells with or without Myc-USP4 plasmid. Co-IP and western blotting results showed that USP4 overexpression strongly inhibited protein poly-ubiquitination (Fig. 5e). Inversely, knocking down USP4 in MHCC97H cells promoted protein poly-ubiquitination (Fig. 5f). Next, we treated the two cell lines with cycloheximide (CHX), a protein synthesis inhibitor, to explore the relationship between USP4 expression and CypA degradation. As the results show, the half-life of CypA was decreased due to USP4 silencing in MHCC97H cells. In contrast, USP4 overexpression in Huh7 cells resulted in an increase in the half-life of CypA (Fig. 5g). Collectively, these results suggested that USP4 regulates CypA stability by preventing degradation through deubiquitination.

### CypA is indispensable for the USP4-mediated promotion of cellular activity and signaling pathway activation in HCC cells

**Fig. 6:**
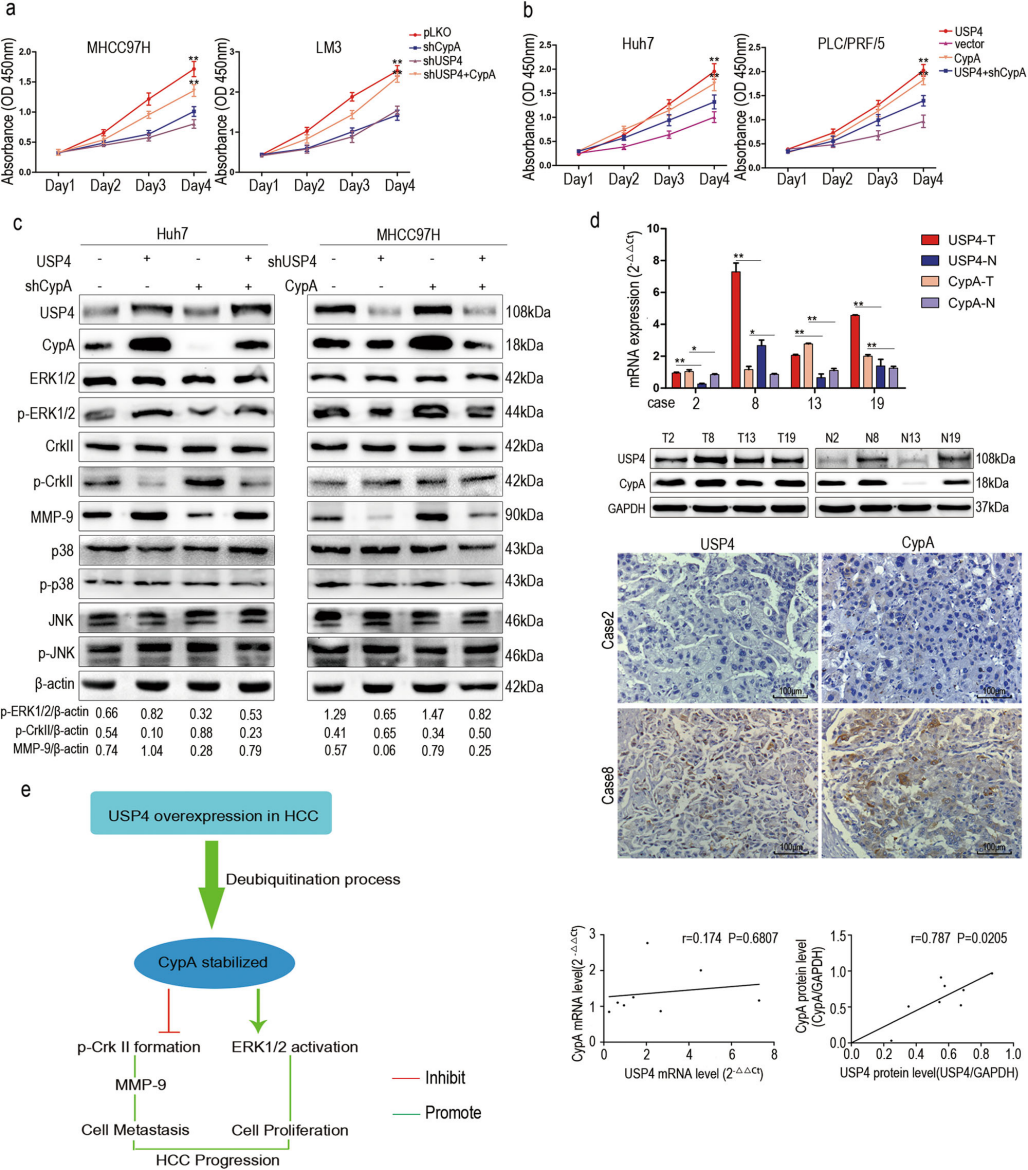
CypA is indispensable for the oncogenic role of USP4. **a**, **b** The effects of CypA on USP4-induced proliferation were investigated in HCC cells using CCK-8 assays. Statistical analyses were determined using one-way ANOVA. **P < 0.01 compared with shUSP4 and shCypA groups (**a**). **P < 0.01 compared with vector and USP4+shCypA groups (**b**). **c** The expression of ERK1/2, p-ERK1/2, CrkII, p-CrkII, p38, p-p38, JNK, p-JNK, and MMP-9 were detected in MHCC97H and Huh7 cells with different forced USP4 and CypA expression levels by western blotting assays. **d** USP4 and CypA expression were detected using qRT-PCR, western blotting analysis and IHC staining in clinical specimens. Representative images are shown. T HCC tissues; N corresponding normal tissues. Spearman correlation analysis was performed on USP4 and CypA levels in HCC tissues at the protein and mRNA levels. Error bars represent the SD. *P < 0.05, **P < 0.01. Scale bars: 100 μm. **e** Schematic representation of the function and potential mechanism of USP4 in HCC progression

CypA is a peptidyl-prolyl *cis–trans* isomerase that has been found to be closely related to cancer development and is involved in many biological processes^20, 21^. Therefore, we supposed that CypA may affect the oncogenic role of USP4 in HCC. To test this hypothesis, CCK-8 assays were used to investigate the effects of CypA on USP4-induced proliferation of HCC cell lines. The results showed that cell proliferation was significantly inhibited by decreasing USP4 or CypA and rescued when CypA was re-expressed in MHCC97H-shUSP4 and LM3-shUSP4 cells (Fig. 6a). Inversely, overexpression of USP4 or CypA in Huh7 and PLC/PRF/5 cells significantly promoted cell proliferation; however, CypA knockdown impaired the effect in Huh7-USP4 and PLC/PRF/5-USP4 cells **(**Fig. 6b**)**. Taken together, CypA is indispensable for USP4-mediated cell proliferation in HCC.

Further, we investigated the mechanism by which USP4 promotes growth and metastasis in HCC cells. Several studies have found that CypA is associated with activation of a variety of cancer-related signaling pathways, including the MAPK pathway^22–25^. In addition, CypA is thought to be involved in tumor metastasis through regulation of CrkII activation, a signaling adapter protein^26^. To determine whether USP4 regulates cell pathways depends on CypA in HCC, we next examined the USP4/CypA complex and related protein levels in MHCC97H and Huh7 cells using western blotting.

The results showed that overexpression of USP4 in Huh7 cells significantly increased the phosphorylated ERK1/2 (p-ERK1/2) protein levels and inhibited the formation of phosphorylated CrkII (p-CrkII), an inactive form of CrkII, but did not affect ERK1/2 and CrkII levels. Subsequently, CypA knockdown alleviated the activation effect of USP4 on p-ERK1/2 and slightly increased p-CrkII in Huh7-USP4-shCypA cells compared with Huh7-USP4 cells. In contrast, inhibition of USP4 by shRNA in MHCC97H cells reduced p-ERK1/2 and increased p-CrkII levels. Similarly, these expression levels were rescued when cells were co-transfected with CypA and shUSP4 plasmids. Similar results were also observed in terms of matrix metalloproteinase-9 (MMP-9) protein levels (Fig. 6c). We did not observe significant changes in p38, phosphorylated p38 (p-p38), JNK, and phosphorylated JNK (p-JNK) protein levels. These data indicate that USP4 positively regulates the ERK1/2–MAPK and CrkII signaling pathways via a CypA-dependent process.

We then examined the relationship between USP4 and CypA in clinical HCC tissues. Consistent with the results in HCC cell lines, there was a positive correlation between USP4 and CypA at the protein level according to western blotting and IHC staining, but not at the mRNA level (Fig. 6d). These clinical tissue data strengthen our presumption that USP4 controls the CypA protein level in patients through deubiquitination, which may indicate that USP4 overexpression, which subsequently regulates CypA protein levels, is a hazard parameter for poor prognosis of HCC patients.

## Discussion

Increasing evidence has confirmed that USP4 plays an important role in tumor progression. However, the function and mechanism of USP4 in cancer development is controversial. Li et al.^27^ found that USP4 was down-regulated in breast cancer tissues and cells, suggesting a tumor suppressor role. Consistent with this, the results of Hou et al.^28^ showed that USP4 promotes apoptosis and inhibits NF-κB activation in head and neck squamous cell carcinoma. In contrast, USP4 has been identified as an oncogene in many studies^11, 13, 29^. However, the roles of USP4 in HCC are still unclear.

In this study, we found that USP4 acts as an oncogene that promotes HCC progression. First, the results showed that USP4 was overexpressed in human HCC tissues compared with adjacent normal tissues. Interestingly, the MHCC97H, SMMC7721, and LM3 human HCC cell lines with higher proliferation and metastasis abilities expressed higher mRNA and protein levels of USP4, which suggested that USP4 may have a role in promoting HCC development. In addition, USP4 expresses higher in HCC cell lines than in the normal liver cell line, LO2. However, whether there is any relationship between USP4 and HCC malignant transformation remains to be determined. Second, clinical data from patients with HCC indicate that USP4 should be considered an independent prognostic biomarker for poor outcome. Additionally, high USP4 expression resulted in a significantly lower OS and DFS in HCC patients. Further, the effect of USP4 on biological function investigated in HCC cell lines showed that USP4 knockdown significantly impaired HCC cell proliferation, colony formation, migration, and invasion in vitro and inhibited tumor growth and intrahepatic metastasis in vivo. Inversely, the biological functions were strengthened or rescued by USP4 overexpression, supporting our finding that USP4 plays an important role in HCC progression.

CypA has peptidyl-prolyl isomerase enzymatic activity, is the most abundant member of the cyclophilin family, and is universally expressed in a variety of cell types. CypA has been shown to play important roles in many biological processes, such as protein folding and trafficking, immune modification, and signaling pathway activation^30, 31^. Recently, increasing evidence has suggested that CypA is overexpressed in many human diseases, including malignant tumors^32^, such as gastric cancer^33^, pancreatic cancer^23^, cholangiocarcinoma^24^, and lung cancer^34^. In cancer progression, CypA modulates multiple signaling pathways, including the NF-κB^35^, Abl-Crk^26^, MAPK, and PI3K/AKT^36^ pathways. In current study, we propose that CypA is indispensable for uncovering the roles and mechanism of USP4 in HCC progression. An important finding in this study is that CypA is regulated by USP4 in HCC cells. The results revealed that USP4 forms a complex with CypA, both endogenously and exogenously, and is responsible for CypA stabilization via deubiquitination. These observations are compatible with the idea proposed in other studies that CypA acts as an oncogenic regulator to promote cancer development^34, 37^, and are consistent with the hypothesis that post-translational modification of CypA is regulated by USP4. In addition, examination of HCC cell lines and clinical samples confirmed that there is a positive correlation between USP4 and CypA protein expression but not mRNA transcription levels. Cell proliferation tests also confirmed the indispensable role of CypA in USP4-mediated cellular activity.

To find the downstream mechanism of the USP4/CypA complex in HCC development, the MAPK and CrkII signaling pathways were chosen to test in the current study.

The Crk (CT-10 regulation of kinase) family consists of a series of adaptor proteins that are universally expressed in tissues. CrkII plays a vital role in signal transduction between non-receptor tyrosine kinases and effector proteins and is involved in cell adhesion, motility, apoptosis, and proliferation by mediating signaling through its SH2 and SH3 domains^38^. Therefore, CrkII is considered a proto-oncogene^39^. Saleh et al. reported that one mechanism by which CypA promotes cell migration is through interaction with CrkII. CypA reduces the inhibited state of CrkII, phosphorylated CrkII (p-CrkII), and upregulates CrkII expression^26^. In our study, results showed that USP4 positively regulates the ERK1/2–MAPK signaling pathway and inhibits p-CrkII via a CypA-dependent process. However, no significant change was seen in the levels of Crk II, which is in contrast with the previous study^26^. We presumed that might be because the USP4/CypA complex does not increase the active form of CrkII directly in HCC cells, but inhibits the formation of its inactive form and disrupts the balance between the two molecules. However, the detailed molecular mechanism of this phenomenon should be explored further in the future.

In conclusion, the current study shows that USP4 is an important protein that facilitates HCC progression. We demonstrated that USP4 drives HCC progression by stabilizing CypA through direct interaction and deubiquitination and thus constitutively activates the MAPK and CrkII signaling pathways (Fig. 6e). These data indicate that USP4 may act as a novel marker for predicting prognosis and may present a therapeutic opportunity for HCC.

## Materials and methods

### Antibodies

Antibodies against USP4 (#2651), ERK1/2 (#4695), phospho-ERK1/2 (#4370), CrkII (#3492), phospho-CrkII (#3491), PARP (#9532), cleaved-PARP (#5625), cleaved-caspase3 (#9661), HA-tag (#3724), Myc-tag (#2276), Flag-tag (#14793), rabbit mAb IgG (#3423), and mouse mAb IgG (#3420) were purchased from Cell Signaling Technology (USA). Antibodies targeting CypA (ab41684, ab58144), MMP-9 (ab38898), and Ki-67 (ab15580) were purchased from Abcam (UK). Antibodies against USP4 (for Co-IP and IHC, sc-376000), p38 (sc-7972), p-p38 (sc-7973), JNK (sc-7345), P-JNK (sc-6254) were purchased from Santa Cruz Biotechnology (USA). Anti-β-actin (AF0003), anti-GAPDH (AF0006), anti-mouse IgG (HRP) (A0216), and anti-rabbit IgG (HRP) (A0208) antibodies were purchased from Beyotime Biotechnology (China).

### Cell lines and culture

The human HCC cell lines MHCC97H, MHCC97L, and LM3 were kindly provided by the Liver Cancer Institute of Zhongshan Hospital, Fudan University (Shanghai, China). The HCC cell lines HepG2, PLC/PRF/5, and Hep3B were purchased from American Type Culture Collection (ATCC), and 293T cells and the HCC cell lines Huh7 and SMMC7721 were purchased from the Cell Bank of the Type Culture Collection of the Chinese Academy of Sciences (Shanghai, China). PLC/PRF/5 cells were cultured in MEM (high glucose, HyClone, USA) supplemented with 10% fetal bovine serum (FBS; Gibco, USA), and all other abovementioned cell lines were cultured in Dulbecco's modified Eagle's medium (high glucose, HyClone, USA) supplemented with 10% FBS. All cells were incubated at 37 °C under a humidified atmosphere with 5% CO_2_.

### Patients and follow-up

Collection of the patient-derived samples was approved by the institutional review boards of Shanghai General Hospital Affiliated with Shanghai Jiao Tong University, and the procedures were executed according to the approved guidelines.

In total, 80 HCC patient specimens were obtained immediately after surgery from December 2008 to September 2012 in Shanghai General Hospital. All patients provided written informed consent. The research was approved by the Biomedical Research Ethics Committee, Shanghai Jiao Tong University, School of Medicine and was carried out according to the World Medical Association Declaration of Helsinki. No patients received any anti-tumor therapies before surgery. Diagnoses were confirmed by at least two certified pathologists, and the tumor stage and grade classification of the specimens were determined according to the International Union Against Cancer guidelines. OS and DFS rates were defined as the interval from the initial surgery to death and clinically or radiologically confirmed recurrence/metastasis, respectively.

### Tissue microarrays, IHC, and evaluation

Tissue microarrays (TMAs) that included 80 pairs of HCC and adjacent normal tissues were constructed, and IHC staining of TMAs was performed as previously described^40, 41^.

The IHC results were judged by two blinded pathologists. IHC staining scores were evaluated according to the percentage and staining intensity as follows: <10% of the cells stained positive (0); 11–25% of the cells stained positive (1); 26–50% of the cells stained positive (2); 51–75% of the cells stained positive (3); and >75% of the cells stained positive (4). The staining intensity scores were graded as no staining (0), light staining (1), intermediate staining (2), and dark staining (3). The formula was used to determine the staining positivity: overall scores = percentage score × intensity score. An overall score of 0–3 was considered negative expression; 4–6 weak expression; and 8–12 strong expression. The following primary antibodies were used: anti-USP4 (1:200), anti-CypA (1:200), and anti-Ki-67 (1:400).

### RNA extraction, qRT-PCR, and western blot analysis

Total RNA was extracted from human HCC cell lines, tumor tissues, and adjacent normal tissues from HCC patients using TRIzol reagent (Invitrogen, USA) according to the manufacturer’s instructions. PrimeScript™ RT Master Mix kit (RR036A, TaKaRa, Japan) was used to reverse transcribe total RNA (500 ng/10 µl reaction volume) according to the manufacturer’s directions. A SYBR® *Premix Ex Taq*™ (Tli RNaseH Plus) kit (RR420A, TaKaRa, Japan) was used for the qRT-PCR assay. The following primers were used for qRT-PCR: GAPDH, forward 5′CCATGTTCGTCATGGGTGTGAACCA3′ and reverse 5′GCCAGTAGAGGCAGGGATGATGTTC3′; USP4, forward 5′CTTTGGTTTGCCCAGAATGT3′ and reverse 5′CAGTGAGGGTCAGCAGGAAC3′; and CypA, forward 5′ GGTCCTGGCATCTTGTCCAT3′ and reverse 5′ AACACCACATGCTTGCCATC3′. Each test was run in triplicate, and the relative target mRNA level was calculated using 2^−∆∆Ct^.

Tissue and cell lysates were extracted using RIPA lysis buffer with the protease inhibitor phenylmethanesulfonyl fluoride (Beyotime Biotechnology, Jiangsu, China). Protein concentration was measured using a BCA protein assay kit (Beyotime Biotechnology) according to the manufacturer’s instructions. Equal amounts of protein (20 μg) were electrophoresed on sodium dodecyl sulfate polyacrylamide gel electrophoresis gels (the concentration of the gel was decided according to the protein molecular weight) and then transferred onto PVDF membranes (Millipore, Billerica, MA). The membranes were blocked in 10% skim milk in TBST buffer for 1 h at room temperature, followed by incubation with primary antibodies at 4°C overnight. After incubation with a secondary antibody for 1 h at room temperature, the proteins were detected using ECL reagent (Millipore).

### Transfection and generation of stable cell lines

A lentivirus-based pLKO vector was used for the RNA interference experiments and generation of stable cell lines. USP4, CypA, and ubiquitin synthetic oligonucleotides were cloned into a pCMV vector (Obio Technology, Shanghai, China). The constructed shRNA and plasmids were transfected into cells using Lipofectamine 2000 (Invitrogen, USA) according to the manufacturer’s instructions. The following target sequences were used: USP4-shRNA1, TGCTAGTGTTCTGTCTAAA; USP4-shRNA2, CTACCGAGGCGTGGAATAA; USP4-shRNA3, TGACTGACTACTTTCTCAA; CypA-shRNA1, GCACTGGAGAGAAAGGATT; CypA-shRNA2, GCATCTTGTCCATGGCAAA; CypA-shRNA3, GCATGTGGTGTTTGGCAAA.

### Transwell migration, Matrigel invasion, and wound healing assays

In vitro migration and invasion assays were performed using a transwell 24-well Boyden chamber (Corning, USA) with 8.0-μm pore polycarbonate membranes without (migration) or with (invasion) Matrigel (BD Biosciences, USA). Briefly, 200 mL of cell suspension (2 × 10^4^/chamber) was added to the upper chambers in serum-free medium and incubated for a minimum of 24 or 36 h. The bottom chamber was filled with 500 mL of medium supplemented with 10% FBS as a chemoattractant. Cells were fixed with 4% paraformaldehyde and stained with 0.1% crystal violet for 1 h after the experiment. Cells were counted in five random fields. Experiments were repeated in triplicate.

Experimental cells were seeded and grown to full confluence in a six-well plate (1.5 × 10^5^ cells/ well). After 12 h, a uniform scratch was made down the center of the well with a sterile pipette tip, and then, the cells were rinsed once with phosphate-buffered saline. Cells were cultured in serum-free medium during the experimental periods. Images were captured in 12-h intervals, and wound widths were quantified and compared to baseline values.

### Cell proliferation and clone formation assays and flow cytometry to evaluate apoptosis

Cell Counting Kit-8 (Dojindo, Japan) was used according to the manufacturer’s instructions to assess cell proliferation, and the results were evaluated by measuring the absorbance at 450 nm on a Gen5 microplate reader (BioTek, USA). Clonogenic assays were performed as previously described^42^. Apoptosis assays were performed using a PE Annexin V apoptosis detection kit (BD Biosciences) according to the manufacturer’s instructions followed by flow cytometry (FACSCalibur; BD Biosciences).

### In vivo tumor growth and metastasis assays

All of the animal experiments were approved by the Institutional Animal Care and Use Committee of the Shanghai Jiao Tong University School of Medicine. Four-week-old male BALB/c nude mice were used and randomly divided into two groups, with six mice in each group. For the subcutaneous tumor growth assay, experimental or control cells (2 × 10^6^/150 μL) were injected subcutaneously into the groin of nude mice. Tumor diameters were measured every 3 days 1 week after the injection. Tumor volume was calculated according to the formula: volume = length × width^2^ × 1/2. Three weeks later, all mice were sacrificed, and tumors were removed and fixed with formalin. The metastasis experiments were performed as previously described^43^.

### Proteomic analysis by LC-MS/MS

Peptides were dissolved in solvent A (2% FA in 3% ACN) and directly loaded onto a reverse-phase Trap column (Chrom XP C18-CL-3 m 120A, Eksigent). Peptide separation was performed using a reverse-phase analytical column (3C18-CL-120, 3 µm, 120A, Eksigent) with a linear gradient of 4–22% solvent B (0.1% FA in 98% ACN) for 50 min, 22–35% solvent B for 12 min, increasing to 80% solvent B in 4 min, and then holding at 80% solvent B for the last 4 min, all at a constant flow rate of 300 mL/min on an Eksigent NanoLC 2D system. The resulting peptides were analyzed with a Triple-TOF 5600+mass spectrometer (AB Sciex).

### Immunofluorescent staining, GST pull-down assay, and Co-IP

Co-IP assays were performed as previously described^11^. In the GST pull-down assays, 293T cells that stably expressed USP4 were lysed. Twenty micrograms GST or GST-CypA fusion proteins (Sangon Biotech, Shanghai, China) and 20 μL GST-beads (Santa Cruz, sc-2009) were incubated with cell lysates for 6 h at 4 °C. The beads were then collected and washed three times with RIPA buffer. The bound USP4 was detected by western blotting.

Immunofluorescent staining assays were performed as previously described^43^. The images were obtained by using a confocal microscope.

### Ubiquitination and cycloheximide chase assays

MHCC97H and 293T cells were transfected with Flag-CypA, HA-ubiquitin, Myc-USP4, or USP4-shRNA. The ubiquitination assay was performed as described previously^44^. A cycloheximide (CHX) chase assay was used to determine the half-life of CypA. After cells were transfected with shUSP4 or USP4 plasmids, they were treated with CHX (100 mg/mL) for the indicated times, and western blotting was subsequently performed.

### Statistical analysis

Three independent experiments were performed for each assay. Statistical analysis was performed with SPSS software (22.0). The results are expressed as the mean ± standard deviation (SD). Student’s *t-*test or Wilcoxon test were used for comparisons between groups. One-way ANOVA was used for multiple groups’ comparisons. The relationships between USP4 expression and clinicopathological factors were determined using Pearson *χ*^2^ test or Fisher’s exact test. Correlation analysis was performed between USP4 and CypA. Survival rates were analyzed using the Kaplan–Meier method and log-rank tests. A hazard ratio (HR) with a 95% confidence interval in the Cox proportional hazard regression model was used to analyze independent prognostic factors. *P* < 0.05 was considered statistically significant. *P < 0.05 and **P < 0.01.

## Electronic supplementary material

Supplementary Figure 1

Supplementary Figure 2

Supplementary Figure 3

Supplementary Figure Legends
