# Supplementary figures and images for: Ubiquitin-specific protease 4 promotes hepatocellular carcinoma progression via cyclophilin A stabilization and deubiquitination
Source: Cell Death Dis. 2018 Feb 2;9(2):148. doi: 10.1038/s41419-017-0182-5 (PMC5833721; doi:10.1038/s41419-017-0182-5)

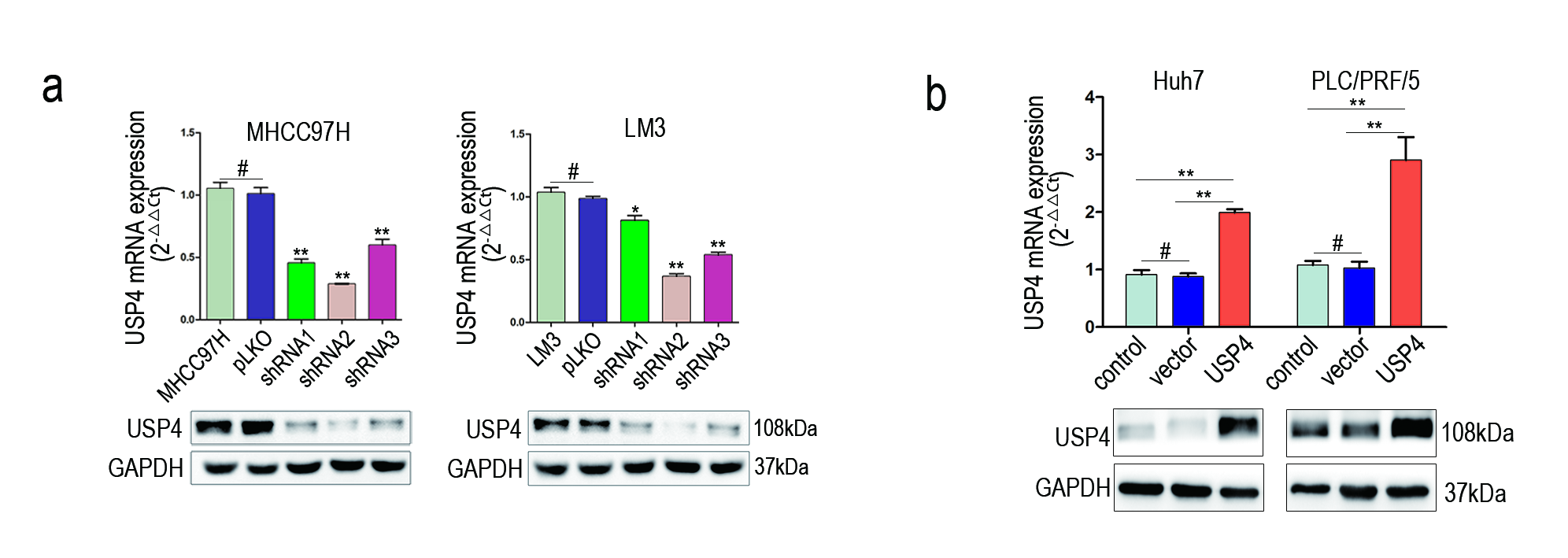

Supplement: Supplementary file 1 — Supplementary Figure 1 [file 41419_2017_182_MOESM1_ESM.tif]

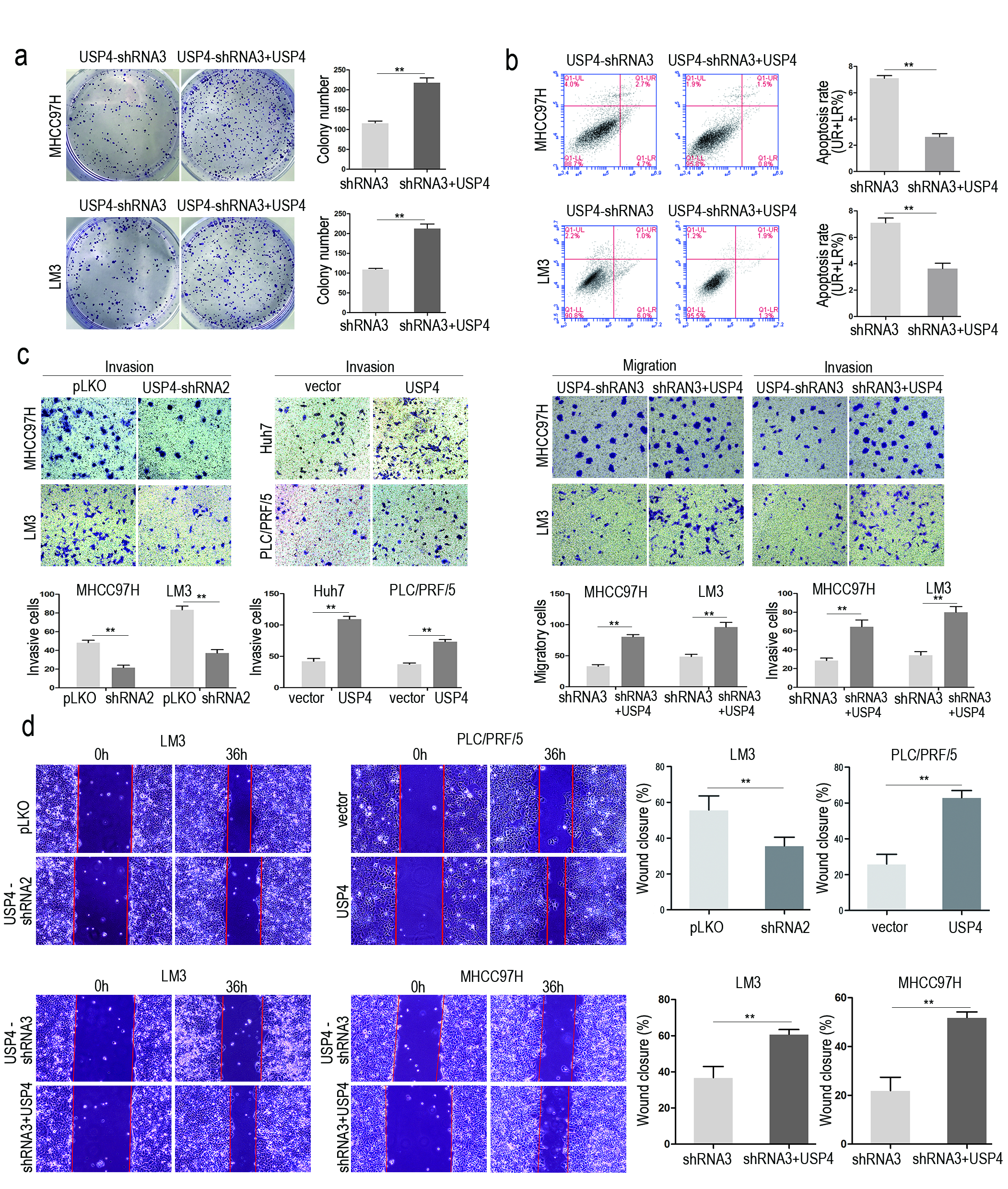

Supplement: Supplementary file 2 — Supplementary Figure 2 [file 41419_2017_182_MOESM2_ESM.tif]

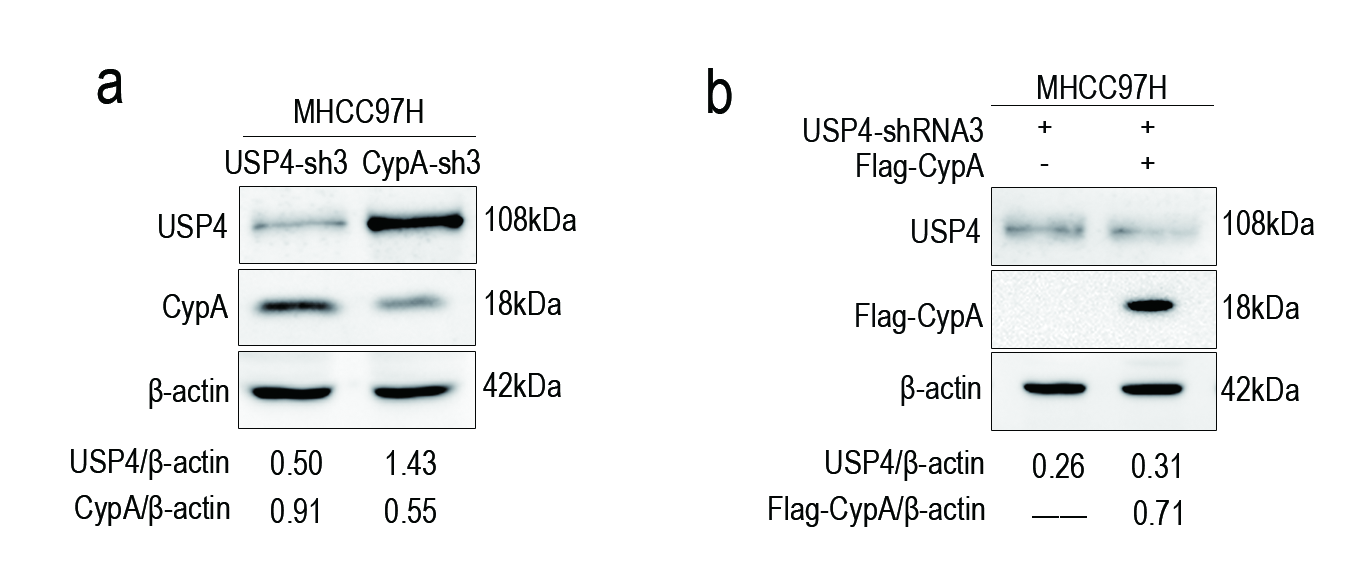

Supplement: Supplementary file 3 — Supplementary Figure 3 [file 41419_2017_182_MOESM3_ESM.tif]
