# Supplementary material for: Ubiquitin-specific protease 4 promotes hepatocellular carcinoma progression via cyclophilin A stabilization and deubiquitination
Source: Cell Death Dis. 2018 Feb 2;9(2):148. doi: 10.1038/s41419-017-0182-5 (PMC5833721; doi:10.1038/s41419-017-0182-5)
Supplement: Supplementary file 4 — Supplementary Figure Legends [file 41419_2017_182_MOESM4_ESM.docx]

**Supplementary figure legends**

**Supplementary figure 1:** (a) (b) HCC cells transfected with USP4-shRNA or USP4 plasmid were established. The transfection efficiency was determined using qRT-PCR and western blotting assays. The statistical analyses compared the experimental group with the control group using one-way ANOVA. Each experiment was repeated three times. Error bars represent the SD. *P<0.05, **P<0.01, and #P>0.05.

**Supplementary figure 2:** (a) Colony formation assays were performed in HCC cells with different forced USP4 expression levels. (b) Apoptosis in HCC cells with different forced USP4 expression was assessed using FCM. (c) The *in vitro* migration and invasion abilities of HCC cells with different forced USP4 expression were assessed using transwell assays. Representative images are shown. (d) Wound healing assays were performed to evaluate the migration of HCC cells with different forced USP4 expression. Cells were cultured in FBS-free medium in the experimental period and wound closure percentage was calculated. Each experiment was repeated three times. Error bars represent the SD. *P<0.05, **P<0.01.

**Supplementary figure 3:** (a) USP4 knockdown affected the protein level of CypA, whereas down-regulated expression of CypA had no effect on USP4 protein level. (b) USP4 knockdown decreased exogenous Flag-CypA protein levels in MHCC97H cells.
